# 

**Published:** 1958-01

**Authors:** 


### Contents

| | | Page |
|---|---|---|
| FOREWORD . . . . . | *Sir Philip Morris* | 1 |
| SOME THOUGHTS OF MEDICAL EDUCATION | *Prof. C. Bruce Perry* | 3 |
| THE MEDICAL LIBRARY OF THE UNIVERSITY OF BRISTOL | *Mr. A. E. S. Roberts* | 12 |
| CARCINOMA OF THE THYROID . . | *Mr. J. W. Bradbeer* | 15 |
| KYPHOSCOLIOSIS WITH PULMONARY HYPERTENSION | *Clinical Pathological Conference* | 22 |
| CORRESPONDENCE . . . . . . . | | 26 |
| NOTES AND NEWS . . . . . . . | | 26 |
| APPOINTMENTS . . . . . . . | | 27 |

